# Successful Transurethral Resection of Recurrent Tumor in an Isolated Bladder Diverticulum Under Transrectal Ultrasound Guidance

**DOI:** 10.1002/iju5.70033

**Published:** 2025-04-15

**Authors:** Yusuke Motoki, Takeshi Sano, Hisanori Taniguchi, Masaaki Yanishi, Hidefumi Kinoshita

**Affiliations:** ^1^ Department of Urology and Andrology Kansai Medical University Hirakata Japan

**Keywords:** isolated bladder diverticulum, transrectal ultrasound guidance, transurethral resection, urothelial carcinoma

## Abstract

**Introduction:**

An isolated bladder diverticulum is a condition where the diverticulum is no longer connected to the bladder lumen. Bladder tumors in isolated bladder diverticula are extremely rare, and treatment methods have not yet been established.

**Case Presentation:**

A 73‐year‐old man who had undergone transurethral resection of a bladder tumor for a papillary tumor near the left ureteral orifice presented with a recurrent tumor within the bladder diverticulum, prompting repeat transurethral resection of a bladder tumor and fulguration of the diverticulum. Although the diverticulum reduced in size, it lost its connection to the bladder lumen. Follow‐up magnetic resonance imaging revealed tumor recurrence within an isolated diverticulum. The tumor was successfully resected via transurethral resection under transrectal ultrasound guidance.

**Conclusion:**

Transrectal ultrasound guidance was useful for transurethral treatment of tumors in isolated bladder diverticulum.

**Summary**

- In cases where bladder tumors develop within an isolated bladder diverticulum, accessing the interior of the diverticulum via transurethral treatment can be challenging.
- Transrectal ultrasound guidance enabled access to the isolated diverticulum by accurately identifying the site of the diverticular orifice incision.

**Abbreviations**
CT: computed tomography
IDBT: intradiverticular bladder tumors
LUTS: lower urinary tract symptoms
MRI: magnetic resonance imaging
TRUS: transrectal ultrasound
TUR: transurethral resection
TURBT: transurethral resection for bladder tumor

## 1 Introduction

A bladder diverticulum is a herniation of the mucosa and submucosa through the detrusor muscle layer, typically resulting from increased intravesical pressure due to lower urinary tract obstruction [1]. Intradiverticular bladder tumors (IDBT) constitute approximately 1% of all bladder tumors and are associated with a higher risk of progression due to the absence of the muscular layer in the diverticulum [2]. Treatment options for localized IDBT are surgical resection, such as open‐, laparoscopic‐, or robot‐assisted laparoscopic radical cystectomy and partial cystectomy, as well as transurethral resection of bladder tumor (TURBT), with or without fulguration of the urothelial mucosa of the diverticulum [3, 4]. An isolated bladder diverticulum refers to a diverticulum that is no longer connected to the bladder lumen.

In this study, we report the case of a patient who developed a papillary tumor in an isolated bladder diverticulum following a previous TURBT and underwent successful TURBT and fulguration of the diverticular mucosa under transrectal ultrasound guidance.

## 2 Case Presentation

A 73‐year‐old male patient presented to a urology clinic with microscopic hematuria. Ultrasonography revealed a 2.5‐cm tumor in the bladder, and cystoscopy revealed a papillary tumor near the left ureteral orifice. The patient was then referred to our hospital for further evaluation and treatment. Computed tomography (CT) showed no apparent upper urinary tract tumors; however, a tumor was detected near the left ureteral orifice. The urine cytology test was negative. The patient underwent TURBT, and the 2.5‐cm papillary tumor near the left ureteral orifice and a 5‐mm daughter papillary tumor were resected. Histopathological examination revealed a low‐grade, noninvasive papillary urothelial carcinoma classified as pTa.

Three months after TURBT, cystoscopy revealed a bladder diverticulum on the left lateral wall containing a papillary tumor. Another TURBT was performed for IDBT, during which the diverticular neck was incised with a bipolar needle electrode to enlarge the opening, after which tumor resection was carried out. Bladder perforation was observed following sampling of the deeper portion of the tumor. The entire diverticular mucosa, including the perforation site, was fulgurated using a roller electrode. Histopathological examination revealed a low‐grade urothelial carcinoma classified as pTa.

**FIGURE 1:**
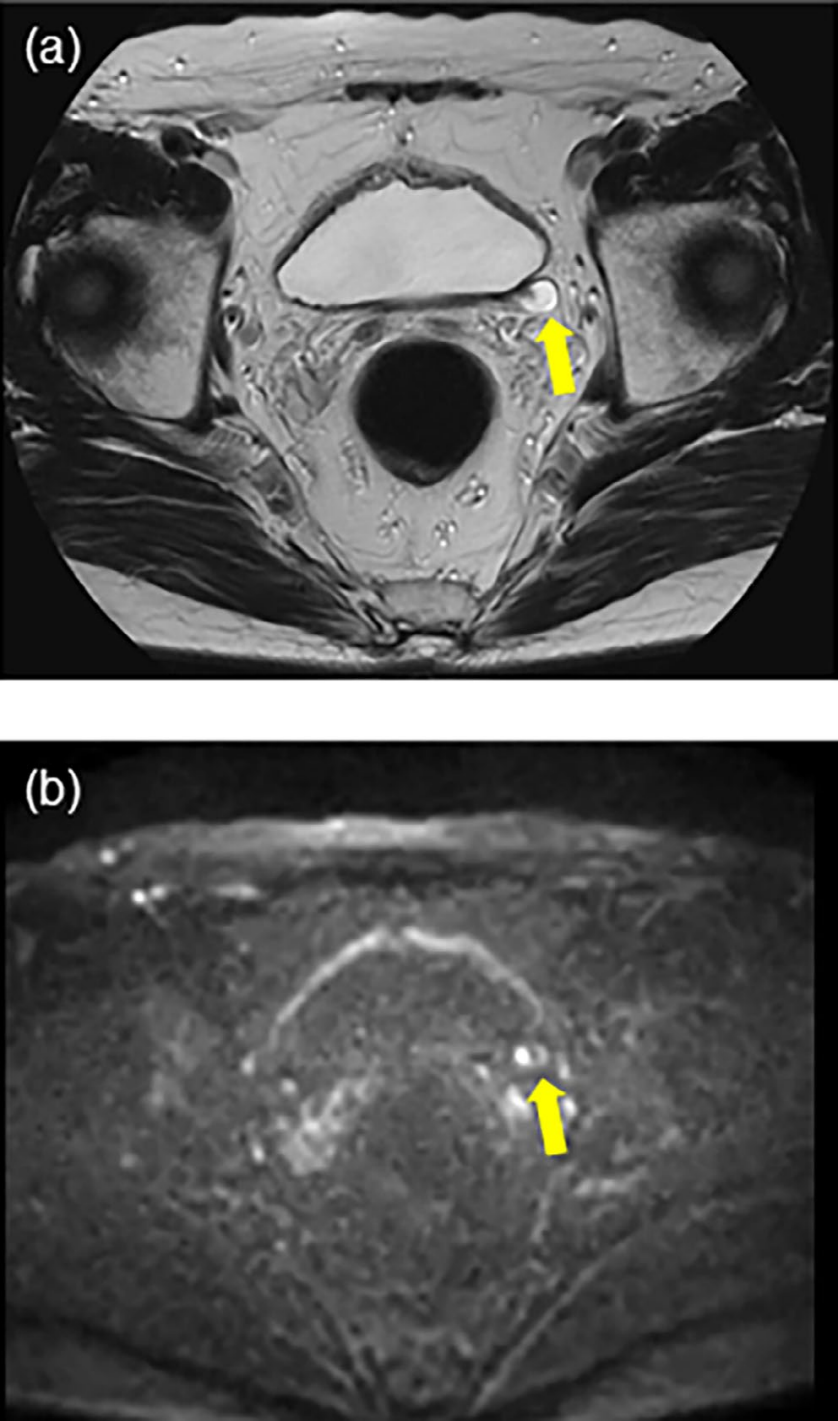
MRI showing bladder tumor in an isolated diverticulum (yellow arrows). (a) T2‐weighted image. (b) Diffusion‐weighted image.

**FIGURE 2:**
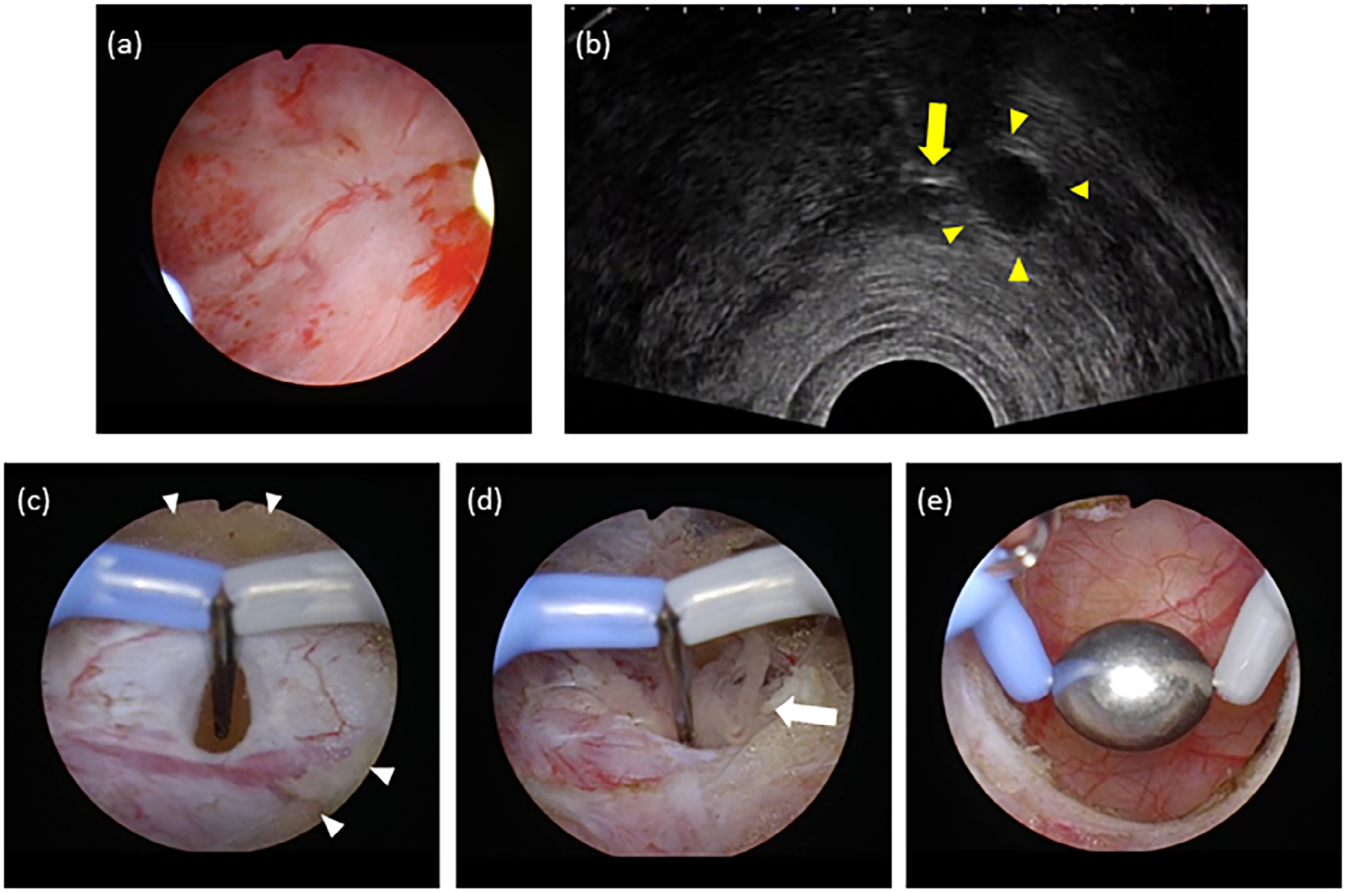
(a) Observation of the bladder lumen shows a flat mucosa behind which the fulgurated diverticulum is located. (b) Transrectal ultrasound findings showing the isolated diverticulum (yellow arrowheads) and the electrode tip (yellow arrow), (c) Incision of the wall of the isolated diverticulum after resection of the fulgurated mucosa. Fat tissues between the diverticulum and the bladder lumen are seen around the diverticulum (white arrowheads). (d) Papillary tumor protruding from the opened diverticulum (white arrow). (e) Lumen of the diverticulum after tumor resection and before fulguration with the roller electrode.

Three months postoperatively, cystoscopy showed closure of the diverticular neck from the bladder lumen, and subsequent magnetic resonance imaging (MRI) revealed an isolated 7‐mm diverticulum. Subsequently, regular follow‐up every 3 months was performed using cystoscopy and MRI. Over three years of follow‐up, no apparent tumor recurrence was observed until MRI detected a 2‐mm tumor within the isolated diverticulum, characterized by low signal intensity on T2‐weighted images and high signal intensity on diffusion‐weighted images (Figure 1). A follow‐up MRI performed three months later revealed an increase in tumor size, prompting another TURBT. We used transrectal ultrasound (TRUS) to localize the diverticulum, which successfully enabled access to the diverticulum by incising the septal wall between the bladder lumen and diverticulum using a needle electrode. The recurrent papillary tumor was resected, and the entire diverticular mucosa was filled with a roller electrode (Figure 2). Histopathological examination confirmed a low‐grade urothelial carcinoma.

**FIGURE 3:**
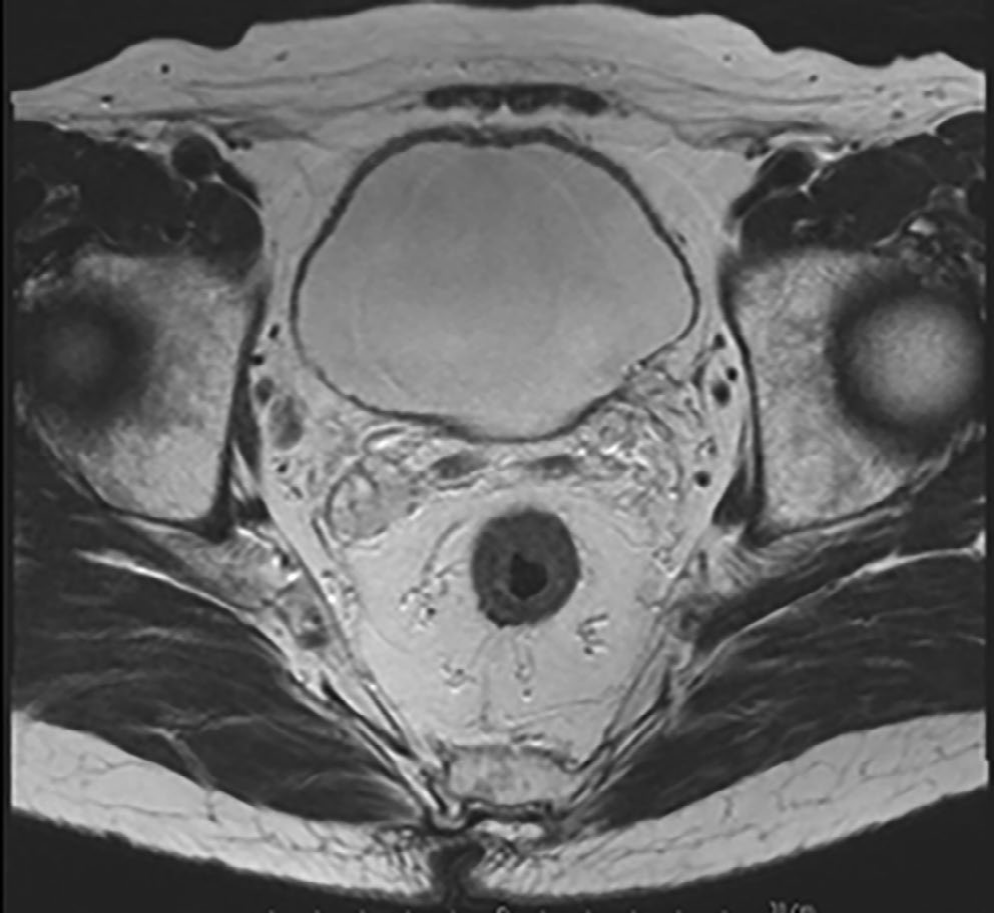
T2‐weighted image of MRI after resection of IDBT and fulguration of the isolated diverticulum.

Three months postoperatively, an MRI revealed a collapsed diverticulum (Figure 3). The patient remained tumor‐recurrence‐free without complications for 10 months postoperatively.

## 3 Discussion

Surgical treatment options for IDBT include TURBT, diverticulectomy, and radical cystectomy. TURBT is recommended for low‐grade, noninvasive, and low‐volume tumors with a broad diverticular neck. However, the thin wall of the diverticulum increases the risk of perforation and tumor seeding [1]. Diverticulectomy is indicated for patients with a narrow diverticular neck and low‐grade, noninvasive, and large‐volume tumors. Radical cystectomy is performed for high‐grade, locally advanced tumors or extensive carcinoma in situ [1, 5].

Herein, we report a patient with a recurrent bladder tumor that developed within an isolated diverticulum and was endoscopically resected. The postoperative follow‐up of IDBT should include abdominal CT or MRI, in addition to cystoscopy, to check for the presence of an isolated diverticulum when flat mucosa is observed instead of an irregular fulgurated mucosa at the site of the original diverticulum. Although a standard treatment for bladder tumors within an isolated diverticulum has not been established because of the lack of previous reports on such conditions, a common and reasonable approach may be diverticulectomy via an extravesical approach due to the difficulty of a transurethral approach. In the current case, because the isolated diverticulum was located near the rectum, TRUS was considered useful for identifying the diverticular neck. Under TRUS guidance, the diverticular neck isolated from the bladder lumen by the septum was efficiently identified and safely reopened, thereby enabling TURBT. This approach may serve as an effective and safe option in clinical scenarios where nonmuscle invasive bladder tumors develop within an isolated bladder diverticulum that can be observed using TRUS.

This study suggests that, in cases where tumor resection via a bladder approach is difficult due to the closure of the diverticular neck, transurethral resection (TUR) under TRUS guidance may be a viable alternative to tumor resection via an extravesical approach.

## Ethics Statement

The authors have nothing to report.

## Consent

Consent was obtained from the patient.

## Conflicts of Interest

The authors declare no conflicts of interest.
